# Mitigating bycatch in Mediterranean trammel net fisheries using species-specific gear modifications

**DOI:** 10.1098/rsos.231058

**Published:** 2023-11-15

**Authors:** Kostas Ganias, Alexandra Karatza, Katerina Charitonidou, Dimitrios Lachouvaris

**Affiliations:** School of Biology, Laboratory of Ichthyology, Aristotle University of Thessaloniki, Thessaloniki 54124, Greece

**Keywords:** small-scale fisheries, trammel nets, elasmobranchs, mitigation measures, sustainable fisheries

## Abstract

Small-scale fisheries (SSF) use static gear which are thought to interact with marine ecosystems more benignly than towed gear. Despite this, trammel nets, one of the most extensively used type of fishing gear in the Mediterranean SSF, generate large amounts of discards, which can account for 25% or more of the captured biomass. Discarded organisms may include endangered or threatened species such as elasmobranchs, as well as non-commercial invertebrates that damage fishing gear or cause disentanglement delays. We evaluated various trammel-net gear modifications, including (i) the use of a guarding net attached to the footrope, (ii) increasing the length of the rigging twine between the footrope and the netting panel, and (iii) decreasing the mesh size of the outer panels. The last two modifications were successful in lowering captures of the marbled electric ray *Torpedo marmorata*, which is commonly discarded in the study area. Both sorts of modifications are relatively simple, their manufacturing does not represent an added cost to implement, and most importantly they do not negatively affect the catch of the target species. The current study shows that prior evaluation of the discard profile of distinct métiers is essential to accomplish species-specific gear modifications and underlines the importance of collaboration among scientists, fishers and gear manufacturers.

## 1. Introduction

The capture and release of unwanted catches is a cause for concern in commercial fisheries because they frequently include undersized individuals of target species as well as other organisms with little to no commercial value, thereby affecting both the yield of fishing activities and the status of wild populations [1,2]. Estimates of discards are required not only to evaluate the impact of fisheries on non-commercial species, but also on ecosystems as a whole [3,4]. At the European (EU) level, discards went to the top of the agenda of the reformed EU Common Fisheries Policy through the establishment of the EU landing obligation [5,6]. However, their evaluation is typically challenging because discard rates vary greatly based on fishing gear and discarding practices, which in turn are determined by landings constraints and economic forces and influenced by environmental and social factors [7,8]. As a general rule, towed gear has the highest discard rates, accounting for the vast majority of discards in global fisheries [9–11]; however, small-scale fisheries (SSF) may also employ gear with significant discard rates [3,9].

In EU waters, the discard ratio for SSF fluctuates because certain organisms, such as elasmobranch and invertebrate species, can be marketed in some regions but discarded in others (e.g. holothurians and batoids which are discarded in [12]). Furthermore, the discard ratio can be influenced by the varying proportion of the catch that is damaged during fishing activity (e.g. fish preyed on during soak or bruised from the net; [13] or by the contribution of biogenic structures such as seagrasses, sponges and corals that are not always included in the discarded biomass [14,15]). For minimizing the discard ratio in SSF, a number of mitigation measures have been proposed, including modifications to gear characteristics and operational and/or post-handling techniques [16–18]. Bottom-set static nets such as gillnets and trammel nets, which are commonly used in SSF, are often subject to modifications that enhance selectivity and reduce bycatch [18]. Modifying the mesh size or hanging ratio, adding an extra panel of non-fishing net to lessen the impact of scavenging organisms, using visual or acoustic cues to deter unwanted by-catch, and avoiding fishing grounds with potentially high unwanted catches are some potential solutions that can be used to reduce unwanted catches in static nets [19]. A big challenge for these modifications is that they must concentrate on reducing the catch of discarded species or organism groups without affecting the catch of commercial species.

One major form of gear modification for lowering discard ratios in bottom-set net catches is to create a no-catch zone for benthic invertebrates to reach the netting panel by minimizing interaction with the seafloor. In trammel nets, this is typically accomplished by placing a strip of gillnet at the bottom of the net, between the footrope and the netting panel. This modification is generally referred to as a *guarding-net* or *selvedge* and has several variations and different names across European regions, e.g. *greca* in Majorca [20], *sardon* and *sardoni* in Turkey and Greece, respectively, *repeu* in Catalonia and so on. This change has been successful in some circumstances (e.g. [19,21,22]), even though in most cases the reduction in discards is accompanied by an equivalent fall in commercial catches (e.g. [23–25]), or even cases where nor commercial catch neither discards were affected by the guarding net [20].

Another type of gear modification involves reducing the capture of flat-bodied bycatch like skates and rays. In recent years, there has been a rise in interest in the incidental capture of elasmobranchs, as they are highly vulnerable to fishing mortality owing to their slow growth, maturity, and limited reproductive potential [26,27]. The majority of these species inhabit coastal habitats [28,29] and have been observed to remain close to the seafloor. That falls within the operational range of SSF thus being impacted primarily by the bottom half of the nets [30]. Gear modification of this type is more related to the technical properties of the netting panel, so that animals would bounce off the net rather than becoming entangled. Thorpe & Frierson [31], for instance, used gillnets with increased tension (larger floats on the head-rope and heavier lead-core lead-line) so that when a shark encountered the gillnet barrier, it would either bounce off the webbing, go straight through the webbing (rather than becoming entangled in it), or be able to manoeuver around the obstruction more easily. Ford *et al*. [32] describe the performance of a modified trammel net with reduced mesh size in the two outer walls, designed to reduce catches of thornback rays (*Raja clavata*); the theory for testing this modification was that thornback rays would bounce off the smaller outer walls and avoid capture. Both studies demonstrated that modified nets have the potential to reduce the bycatch of elasmobranchs; however, these modifications must take into account the species' profile of both the commercial catch and discards, as differences in body size and morphology are the primary determinants for the interaction of captured animals with fishing gear [33].

Trammel nets, one of the most commonly used fishing gear in the Greek SSF, produces high volumes of discards that can account for 25% or more of the captured biomass [10,34,35]. Discarded organisms can include endangered or threatened species like elasmobranchs, as well as non-commercial invertebrates that damage fishing gear or delay disentanglement. We tested various gear modifications in trammel nets including (i) the use of a guarding net fitted to the footrope, (ii) an increase in the size of the rigging twine between the footrope and the netting panel, and (iii) a reduction in the mesh size of the outer panels; all these types of modifications are relatively simple and its manufacturing does not represent an added cost. Our study demonstrates that prior evaluation of the discard profile of various métiers is required to achieve species specific gear modifications and emphasizes the importance of collaboration among scientists, fishers, and gear manufacturers.

## 2. Material and methods

**Figure 1.**
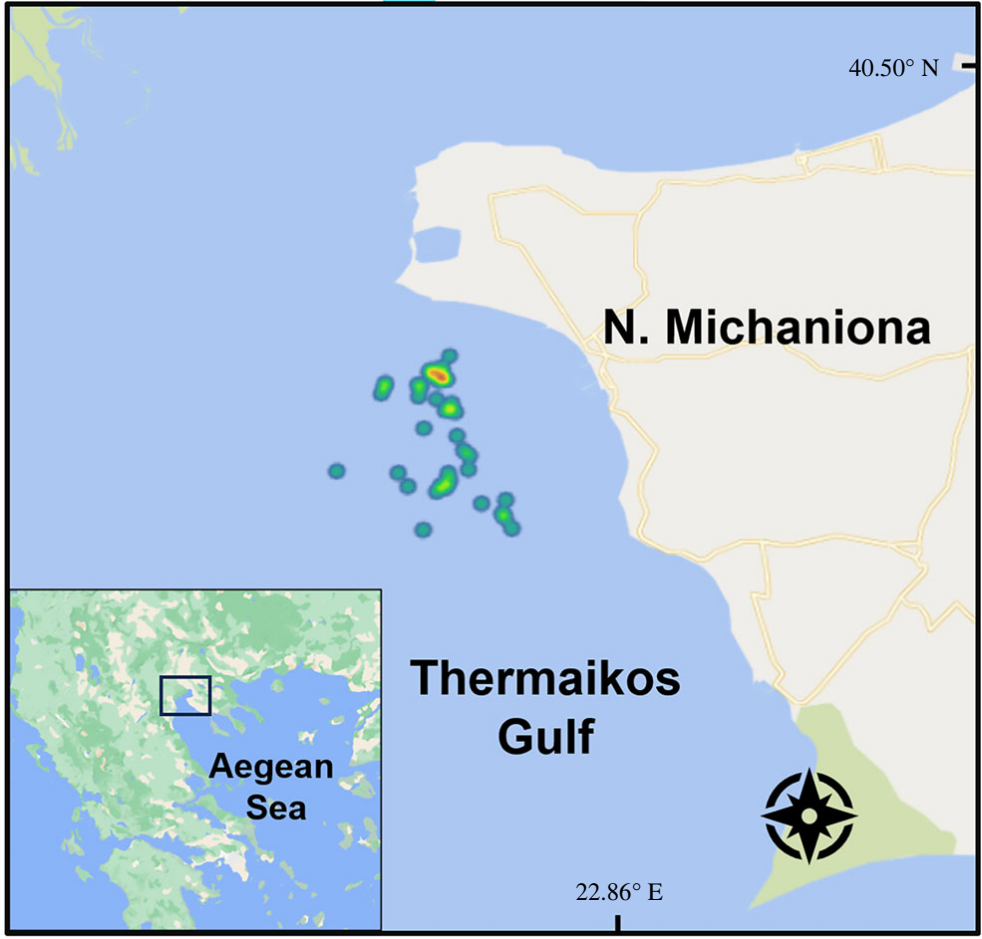
Map of the survey area indicating the locations (heat-map) of the commercial fishing efforts.

Our research was carried out off the coast of Nea Michaniona in the eastern Thermaikos Gulf, one of the most important fishing grounds in the north Aegean Sea (figure 1) and comprised two surveys done over two years. The surveys took place between 25 and 40 m where the composition of the bottom was mostly sand and mud. The first-year survey was conducted under realistic fishing conditions (*commercial survey*) with the goal of providing a comprehensive image of the trammel net catch profile in the surveyed area, i.e. the predominant species caught and the fraction of discards per category of organism groups. During the second year, we examined the catch profile of modified trammel nets, designed to reduce the capture of the discarded bycatch recorded during the commercial survey (*experimental survey*).

### 2.1. Commercial survey

The commercial survey was conducted over a period of seven months, from May to November 2021, with the commercial vessels ‘*Agios Georgios’* (8 m, 15 hp) and ‘*Pantelis*’ (9.2 m, 95 hp). Both vessels deployed trammel nets of 1500–2500 m length, with inner panel mesh size between 32 and 42 mm (knot-to-knot; this is how mesh size will be expressed from now on) and outer panel mesh size was always 5× longer than inner panel mesh. The fishers were primarily targeting the common sole, *Solea solea*; this particular métier, locally known as *glossodychto* (sole-net), takes place at greater depths (35–40 m) between March and October. During each fishing effort, one trammel net was set in the morning and hauled back the following morning shortly after sunrise. A total of 41 fishing efforts were recorded during the course of our study.

In each fishing effort, at least one scientific observer was present to record the geographical coordinates, depth and soak-time, i.e. the time the fishing gear was active in water, and to ensure that all the commercial and discarded biomass, except from seagrass and algae, was recorded. In addition to the commercial and discarded catch the observers also recorded the catches that were kept by the fishers for ‘self-consumption’. These were almost always low-quality captures consisting of damaged individuals or low-value species. Fish removal and net cleaning were performed by the fisher either at sea (most cases) or shortly after the boat returned to the fishing harbour, based on weather conditions and catch quantity. Items from the commercial catch were identified and measured for total weight (TW; g) and total length (TL; mm) in the harbour. When alive, discarded elasmobranchs were returned to the sea, after they were measured and identified, in the best possible state. The remaining discards were placed in a portable cooler and transported to a nearby laboratory for further processing. All individuals were identified to the lowest taxonomic level possible and measured for TW. All organisms, commercial and discards, were grouped into five major groups: teleosts (OSTEI), chondrichthyans (CHON), cephalopods (CEPH), crustaceans (class malacostraca; CRUST) and the remaining taxa of invertebrates (gastropods, echinoderms, corals etc.; INVERT). The catch per species or per group of organisms was expressed as the biomass (CPUE) or abundance (APUE) per fishing effort standardized to the mean gear length (1900 m) and mean soak time (19 h).

### 2.2. Experimental survey

**Figure 2.**
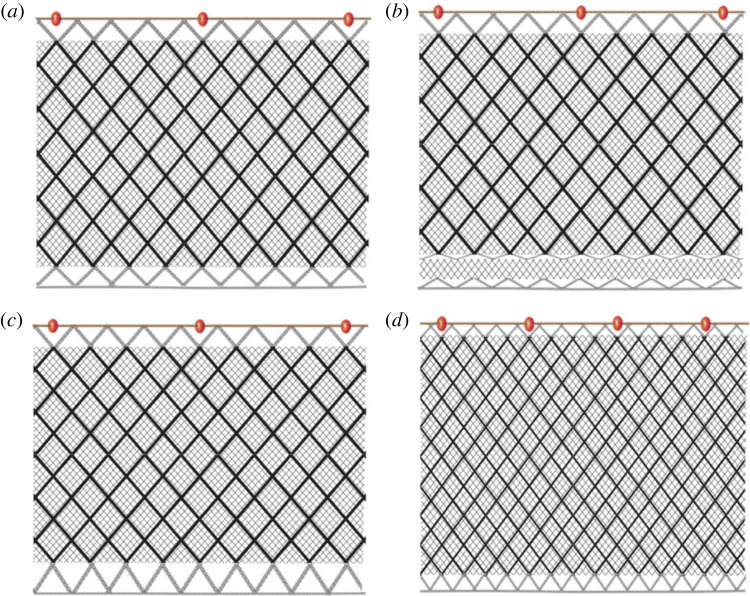
Characteristics of the trammel nets used in the experimental survey, each of which consisted of six 50 m long panels for a total length of 300 m. (*a*) The standard trammel net with 40 mm inner meshes and 200 mm outer meshes. (*b*) The SELV type with a guarding net (gillnet, 40 mm polyethylene mesh, 2.5 mesh height) fitted between the main net and the footrope. (*c*) The ARCH type with double rigging twine (staple) compared to the standard net between the netting and the footrope. (*d*) MESH type, with half the size of the outer mesh compared to the control nets (100 mm instead of 200 mm).

The experimental survey was also conducted off Nea Michaniona with the commercial vessel *Agia Paraskevi*, an 8 m decked polyester boat with 43 hp engine during the period July–September 2022 to the same depths and areas as the commercial survey the year before. The study compared three modified trammel nets to standard trammel nets which were used as control (figure 2 and table 1). The characteristics of the standard nets were similar to the ones used for the commercial survey (figure 2*a*). The first modified gear (SELV; figure 2*b*) was a standard trammel net with a guarding net (gillnet, 40 mm polyethylene mesh, 2.5 mesh high) fitted between the net and the footrope. The theory for assessing this modification was that gillnets only rarely catch rays, at least in the surveyed area [36], and because the selvedge is known to create a barrier zone for benthic scavengers to reach and get entangled by the trammel net (see references in the Introduction). The second modification (ARCH; figure 2*c*) was again a standard trammel net which was fastened to the footrope using the double rigging twine (staple) length compared to the standard net, thus increasing the distance between the netting panel and the seabed (figure 2*c*). The theory for assessing the ARCH was to create escape outlets for benthic rays and a barrier zone for scavenging invertebrates. The only difference between the third modification (MESH; figure 2*d*) and the standard net was the half mesh size of the outer panels (100 mm instead of 200 mm). In such a way flatter animals such as batoids would probably bounce off the webbing instead of being entangled.

**Table 1.** Detailed characteristics of the trammel nets used in the experimental survey.

net/panel	characteristic	control	MESH	ARCH	SELV
whole net	hanging ratio	0,5	0,5	0,5	0,5
	number of floats	55	55	55	55
	length (m)	50	50	50	50
	ringing twine (mm)	100	100	200	100
	footrope (g m^−1^)	110	110	110	110
inner panel	mesh size (mm)	40	40	40	40
	net type	PA, 2010d/3	PA, 2010d/3	PA, 2010d/3	PA, 2010d/3
	*n* meshes	25	25	25	25
outer panel	mesh size (mm)	200	100	200	200
	net type	PA, 2010d/6	PA, 2010d/6	PA, 2010d/6	PA, 2010d/6
	*n* meshes	5	8	5	5
guarding net	mesh size (mm)				40
	net type				PA, 2010d/9
	*n* meshes				3

All nets, both standard and experimental, were rigged in single trammel nets composed of six panels of 50 m per set and each trammel net (300 m in total) was composed of a single type of tested nets. Each fishing trial compared one 300 m experimental net to one 300 m control net. To avoid time/area bias, fishing trials consisted of deploying experimental nets along with control standard nets at adjacent sites. A total of 17, 15 and 14 trials were conducted for SELV, ARCH and MESH nets, respectively. During each trial, the two separate trammel nets were marked with different coloured floats so that each net could be recognized as they arrived aboard. Fishing trials were conducted using the same fishing tactics as the ones used during the commercial survey, and nets were retrieved, cleaned on board and cast for the following fishing trial in a single trip. Because soak times and net lengths were consistent across trials, no additional standardization of the fishing effort was required, and catch per unit effort was expressed as the biomass (CPUE) or abundance (APUE) of various catch categories (e.g. commercial versus discarded; species group or species). The commercial and discarded catch were processed in the same manner as the experimental survey.

All data processing and analyses were conducted in R, version 4.3.1 [37]. A set of generalized linear mixed-effects (GLMM) models were constructed for each of the five organism groups and three types of modified nets to examine the effect of modified nets on the CPUE using the lme4 package. The type of net (experimental versus control) was included as a fixed effect (two-level) while fishing hauls were included as random effects in each model. The DHARMa software was used to inspect residuals and to ensure that models met normality assumptions and overdispersion tests [32].

## 3. Results

### 3.1. Commercial survey

The total amount of biomass collected by the 41 commercial fishing efforts was 756.2 kg. Unidentifiable biogenic material including dead colonies of the coral *Cladocora caespitosa* (5.5 kg) were excluded from the above calculation.

**Figure 3.**
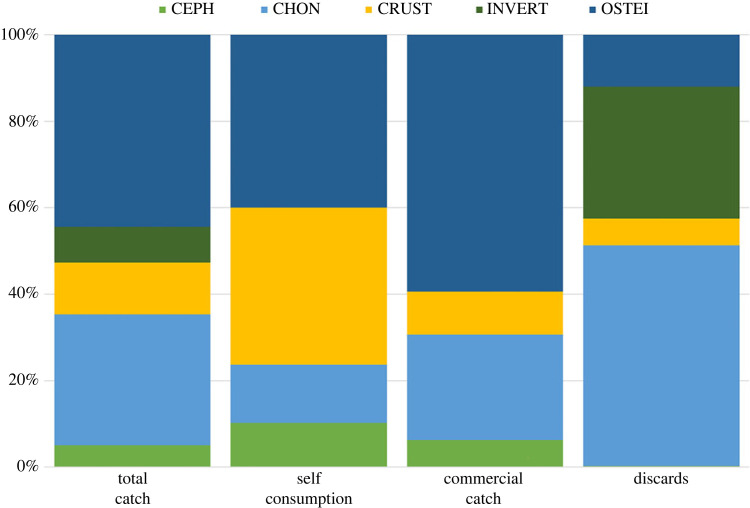
Breakdown of the fraction of different organism groups per catch category in the commercial survey. OSTEI, teleosts; CHON, chondrichthyans; CEPH, cephalopods; CRUST, crustaceans; INVERT, invertebrates.

**Figure 4.**
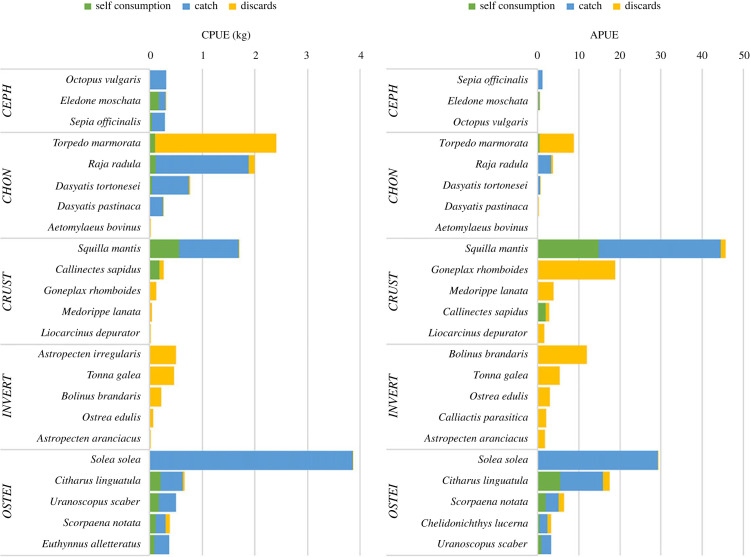
Catch per unit effort (CPUE) and abundance per unit effort (APUE) of the five most captured species per organism group. For each species, the breakdown of the CPUE and APUE per catch category (self-consumption, commercial catch and discards) is also shown. OSTEI, teleosts; CHON, chondrichthyans; CEPH, cephalopods; CRUST, crustaceans; INVERT, invertebrates.

Teleosts were the most important group of organisms both in terms of total biomass (44.8%; figure 3) and total abundance (39.7%). The most abundant teleost captured was the common sole *So. solea*, which accounted for 46.8% of the total teleost biomass while the spotted flounder *Citharus linguatula*, another flatfish, was the second most important teleost in terms of both biomass (8.4%) and abundance (22.3%) (figure 4). The scorpion fish *Scorpaena notata*, the stargazer *Uranoscopus scaber* and the tub gurnard *Chelidonichthys lucerna* were also among the most frequently captured teleosts representing 7.2%, 4.2% and 4.1% of total teleosts abundance, respectively (figure 4).

Trammel nets were extremely selective for elasmobranchs, which accounted for 30% (225.2 kg; mean CPUE = 5.5 kg) of the total biomass (figure 3). Except from one specimen of *Mustelus mustelus* all elasmobranchs were batoids; *Torpedo marmorata* was the most abundant species (CPUE = 2.4 kg, APUE = 8.8), followed by *Raja radula* (CPUE = 1.9 kg, APUE = 3.4), *Dasyatis tortonesei* (CPUE = 0.8 kg; NPUE = 0.7) and *Dasyatis pastinaca* (CPUE = 0.3 kg; NPUE = 0.1) (figure 4); one specimen of *Aetomylaeus bovinus* was captured throughout the entire survey.

Crustaceans, which constituted 12.2% and 37.1% of total biomass and abundance, respectively (figure 3), were also selectively captured by trammel nets. The most important species in terms of biomass and abundance was the mantis shrimp *Squilla mantis* while the highly abundant crabs *Goneplax rhomboides* and *Medorippe lanata* only made up a small portion of the biomass owing to their small body size (figure 4).

Invertebrates made up just a small percentage of total biomass (7.9%), with the sea star *Astropecten aranciacus*, and the gastropods *Tonna galea*, and *Bolinus brandaris* being the most important species. During the commercial survey, only three cephalopod species were captured (figure 4): the common cuttlefish *Sepia officinalis*, the musky octopus *Eledone moschata* and the common octopus *Octopus vulgaris* (figure 4). However, their captures were negligible, accounting for only 5.1% of the total biomass (figure 3).

The commercial catch accounted for 470.1 kg of the total biomass, 91.9 kg was retained by the fishers for self-consumption and 194.2 kg was discarded; therefore, the total discard ratio for trammel nets was 25.3%. The batoids comprised 48.5% of the total discarded biomass, making them the most discarded group of organisms (figure 3). *Torpedo marmorata* was the most discarded species (figure 4) because it was the only batoid with no commercial value, and hence almost all the captured specimens were discarded; only 6% of total items caught were retained by the fishers for self-consumption. All other species of batoids were commercialized, and fishers discarded only those that were smaller than the minimal landing size; the discard ratios for *R. radula*, *D. tortonesei* and *D. pastinaca* were 11.8%, 16% and 16.7%, respectively.

Among the most commonly caught teleosts, *Sc. notata* and *Ci. linguatula* had the greatest discarding ratios (19.1 and 4%, respectively; figure 4), with their very small size being the primary cause for being discarded. During the commercial survey, the two most economically important species, *So. solea* and *Sq. mantis*, exhibited a very low discard ratio (less than 1%), which was almost always owing to damaged specimens from species such as *G. rhomboides* which was the most abundant discard species. However, a high proportion of the biomass *Sq. mantis* (32.9%) was kept by the fishers for self-consumption. All items from the groups of invertebrates caught during the commercial survey were discarded.

### 3.2. Experimental survey

**Figure 5.**
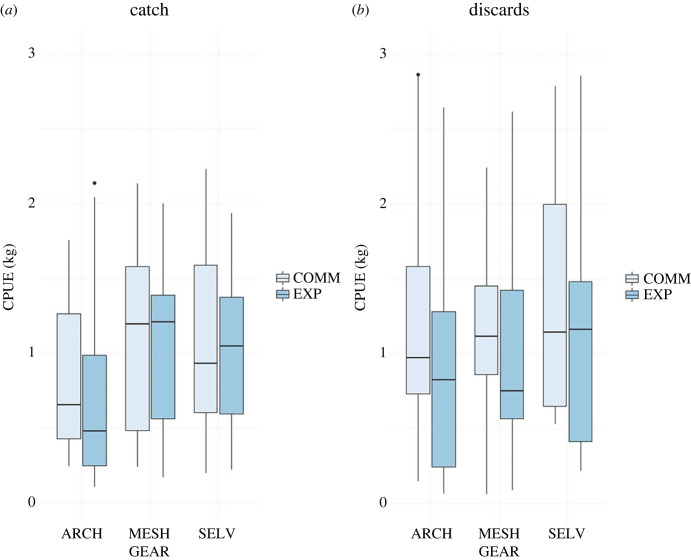
Box and whisker plots for the comparison of the catch per unit effort (CPUE) between the control (COMM) and the modified (EXP) nets for the three different types of modified trammel nets (ARCH, MESH, SELV). The results are shown separately for the commercial catch (*a*) and discards (*b*). Boxes represent the first quartile, the median, and the third quartile, and whiskers represent the lowest and highest data within 1.5× the interquartile range of the first and third quartiles.

In terms of both discarded and retained biomass, GLMM analysis indicated significant variations in how the three types of experimental gear differed from their controls (figure 5). Regarding discards, only the MESH exhibited a significant reduction in biomass (−32.5%; *p* < 0.01); the putative reductions in the ARCH (−16.3%) and SELV (−14.3%) were not significant (*p* > 0.1). In terms of retained catch, none of the examined nets demonstrated a significant difference in biomass. In this sense, the MESH was the only experimental net that was successful in reducing discarded biomass while maintaining retained catch.

The capture of different groups of organisms and species differed between the three types of experimental gear. Regarding batoids, the MESH was the only effective net that reduced their captures by 38.8% (*p* < 0.1). The ARCH showed a 37% decrease that was not statistically significant, while the SELV showed no significant changes. When each batoid species was taken into consideration separately, the picture was more accurate. In the case of *Tor. marmorata*, MESH and ARCH were successful in reducing their captured biomass by −57.8% (*p* < 0.05) and −65.5% (*p* < 0.05), respectively, whereas the SELV had no effect on its captured biomass. On the other hand, none of the modified nets had any effect on the catches of *R. radula* and *Daysatis* sp.

In the case of teleosts, the three types of experimental gear had no significant effect on either their overall biomass or the biomass of *So. solea*, the most important species. The ARCH, on the other hand, dramatically reduced crustacean biomass by 43.4%, owing mostly to a 46.7% decline in *Sq. mantis*, the second most important commercial species after *So. solea*. The other two experimental nets did not make a significant impact on total crustacean biomass or *Sq. mantis* biomass. Finally, none of our tested nets caused any significant change in the caught biomass of invertebrates, which were the other major group of discards.

## 4. Discussion

The current study assessed the efficacy of three different modified trammel net designs in terms of biomass and species composition of commercial catches and discards. It demonstrates that prior evaluation of the discard profile of various métiers is required to achieve species specific gear modifications and emphasizes the importance of collaboration among scientists, fishers, and gear manufacturers. Gear modification is an important mitigation measure for fisheries and métiers that suffer from high proportions of unwanted catch [18], such as the trammel nets targeting common sole in the eastern Thermaikos Gulf, where the present study estimated a discard ratio of 25.3%. In the same region and using the same methodology, Ganias *et al*. [34] estimated the discard ratio for the cuttlefish trammel net fishery to be 25.5%, which is almost equal compared to the métier examined in this study. However, in the cuttlefish fishery, the main group of discards were the invertebrates, whereas in the present study the most important group of discarded organisms were the elasmobranchs. As a result, it is evident that in order to adopt appropriate mitigation measures and/or make relevant gear modifications, not only the discard ratio but also the composition profile of the discarded organisms must be known.

Batoids, especially the marbled electric ray *Tor. marmorata*, constituted the vast majority of discards in the current fishery. Two other species of batoids, the common stringray *D. pastinaca*, and especially the rough ray *R. radula* displayed high CPUE but low discard ratio. Tiralongo *et al*. [38] reported remarkably similar results for trammel nets targeting common cuttlefish in South Sicily, where batoids of the genera *Torpedo*, *Raja*, and *Dasyatis* were again the three most prevalent discards. Concerning *Tor. marmorata*, fishers of the Thermaikos Gulf discard all items captured regardless of body size because this species is not sold in local markets (see also Chatzispyrou *et al*. [39]). In order to avoid being electrocuted, it is common practice for fishers to kill the animals with a hammer or a stone before returning them to the water. Owing to their dangerous defensive behaviour and venomous spine, specimens of *Dasyatis* spp. are also killed prior to net cleaning following landing. In this regard, almost all discarded items of these species have no hope of survival when they are returned to the ocean. In contrast to other areas where all rays, regardless of size, are discarded (e.g. South Siciliy; [38]), *Dasyatis* and *Raja* specimens that exceed the minimum landing weight of 300 g are typically kept and sold by SSF of the Thermaikos Gulf. In other regions, such as in central Portugal [40,41], south Portugal [42], and the Balearic Islands [20], the rays are once again a bycatch of trammel nets, but they are occasionally commercialized depending on fishers' revenue.

**Figure 6.**
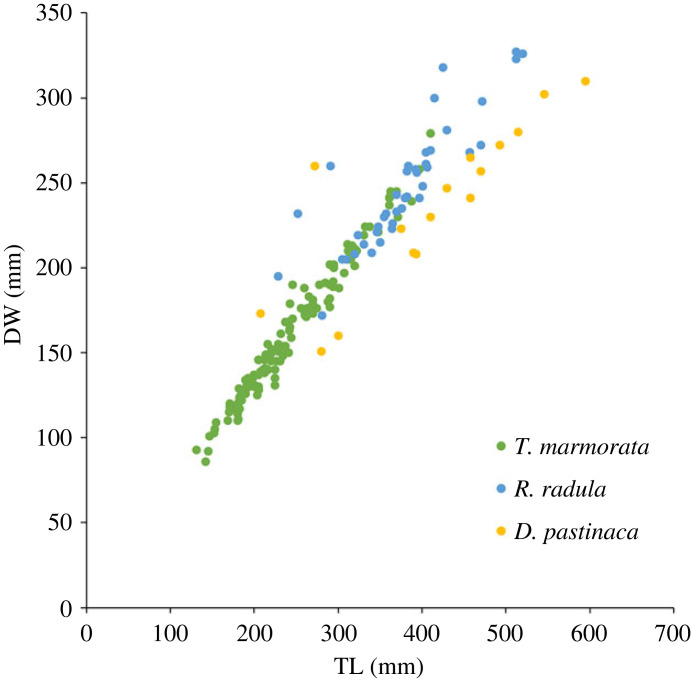
Relationship between total length (TL) and disc width (DW) for *Torpedo marmorata*, *Raja radula* and *Dasyatis pastinaca* caught during the commercial survey.

The trammel nets with the reduced mesh size in the outer walls (MESH) and those with the double staple length at the footrope (ARCH) were the most successful gear modifications in our experimental survey as they both managed to significantly reduce the catch of *Tor. marmorata* by 58% and 66%, respectively, without affecting the catch of *R. radula* and *D. pastinaca.* In our study area, the former species is nearly entirely discarded, whereas the two other species are both commercialized. In MESH, the difference was attributed to the distinct body morphology of *Tor. marmorata*, in which the pectoral fin disc is nearly circular with a straight edge at the very front, whereas the two other species have sub-circular bodies, angular pectoral fins, and either a curved (*R. radula*) or pointed (*D. pastinaca*) snout. Therefore, owing to their pointed bodies, rays and stingrays can easily penetrate the netting bag of a trammel net, whereas *Torpedo* would have a greater chance of bouncing off the net owing to the smaller mesh of the outer panel. The mitigating method in the ARCH is quite different, as it creates an escape zone for mobile benthic species between the netting panel and the seafloor; the escape apertures were sufficiently large to allow smaller batoids to pass beneath the trammel net without becoming entangled. A similar modification is reported for ground gear trawl by Fakıoğlu *et al*. [30]. In fact, *Torpedo* in the commercial survey had smaller bodies than the other two rays (figure 6), with a significant portion of the captured population below 300 mm total length, and 200 mm disc-width which is the utmost aperture width in the ARCH. By contrast, almost all rays had disc-widths greater than 200 mm, reducing their chances of escaping through the apertures in comparison to *Torpedo*. This explains why the *Torpedo* was the only batoid in this net type with a reduced capture rate.

To the best of our knowledge, the two tested types of gear of the present study are the first effective use of trammel-net technical modification for minimizing the bycatch of batoids, or elasmobranch species in general. Ford *et al*. [32], evaluated a comparable modification with smaller mesh at the outer walls, reporting an increase in common sole catches without affecting the catches of the thornback ray; the authors deemed this adjustment a success because it took significantly less fishing effort to catch the same quota of common sole with no increase in thornback ray catches. In the current study, both the MESH and the ARCH were effective in diminishing the catches of a discarded batoid while having no effect on the catches of the target flatfishes *So. solea* and *Ci. linguatulus*. However, the ARCH reduced the catch of the mantis shrimp *Sq. mantis* by 47% which is another target species in the examined fishery. Therefore, taking all these into account the most successful modification was the MESH since it was the only net that mitigated the catches of *Tor. marmorata* without affecting the catch of the target fish and crustacean species.

**Figure 7.**
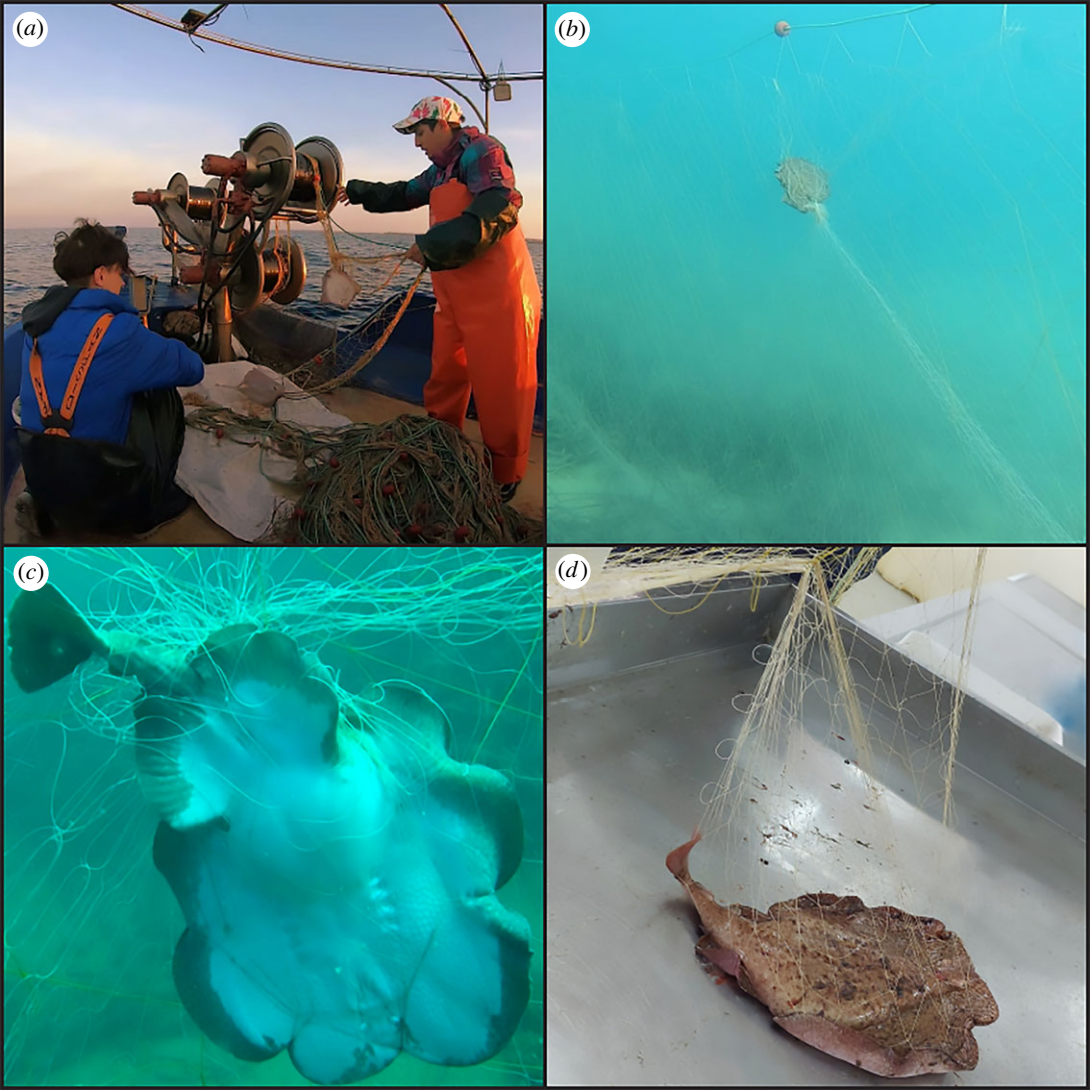
On board (*a*) and *in situ* (*b*) observation showing batoids captured in the middle of the netting panel. The entanglement mechanism of batoids was studied both through *in situ* (*c*) and laboratory (*d*) observations.

Our third experimental net, which was fitted with a guarding net between the netting panel and the footrope, was ineffective at reducing batoid captures. This contradicts our initial premise because a recent study conducted by our team [36] show that local batoids are not trapped by gillnets, leading us to believe that the guarding net, which is a short strap of gillnet, would prevent the capture of these species. However, it appears that instead of leaping off the webbing of the guarding net, batoids swim upwards to avoid this impediment, becoming caught in the trammel net. This was clearly confirmed both by on board (figure 7*a*) and field (figure 7*b,c*) observations of batoids trapped in the middle of the net (figure 7) during their attempt to leap over the netting panel, even though these species generally appear at the bottom part. Field observations of entangled specimens (figure 7*c*) were compared with laboratory observations on the precise capture mechanism of these fishes (figure 7*d*), revealing that larger specimens were captured in larger bags with a larger net surface and, therefore, more meshes, making it more difficult to escape after entanglement.

Currently, the use of a guarding net in trammel net fisheries is almost entirely focused on reducing the capture of benthic scavengers [19]. However, the fraction of benthic invertebrates of the total catch in the current study was fairly low (about 10%), and so the lack of success of all types of modified nets in mitigating their catch had no impact on the surveyed métier. As stated in the Introduction, the use of the guarding net has been ambiguous since, with the exception of a few situations where it reduced discards without affecting catch [19,21], in most cases it lowered both commercial catch and discards [23–25], while in some cases it had no effect on catch at all [20]. We hypothesize that variations in guarding-net efficiency are mostly caused by the species profile of the discards. Also, the composition of the benthic community is a considerable factor for certain species’ presence on the nets. The contribution of murecid gastropods *B. brandaris* and *Hexaplex trunculus*, for example, was relatively modest in the current survey, in contrast to their significant abundance in the cuttlefish trammel net fishery [34], which occurs at lower depths (between 5 and 8 m) where these species are more prevalent. Preliminary findings indicate that, in contrast to the current study, the employment of a guarding net in trammel nets targeting cuttlefish significantly reduces the catches of discards, which, as previously stated, primarily comprise murecid gastropods. In the current investigation, however, invertebrates were predominantly larger bodied organisms like sea stars and the giant tun that were fished with the sweeping action of the net on the seafloor.

The current study indicates that in order to reduce discards in SSF, gear modifications should be preceded by a thorough investigation of not only the amount but also the species composition of the unwanted catch. In our case, for example, the use of a guarding net, which could be advantageous in fisheries with a high concentration of scavenging organisms, was not a successful modification. A preliminary (commercial) survey, on the other hand, revealed that the most appropriate gear modifications for the current métier should focus on reducing batoid by-catch. Indeed, two of the modifications assessed and proposed by our study were successful in reducing the catch of *Tor. marmorata* which was the most discarded organism in the present study. To protect fragile elasmobranchs, additional species-specific strategies and management plans developed in partnership with local fishers are required to introduce modified nets as a way to reduce discard rates. Reluctant fishers may be persuaded to update their current fishing gear because the proposed modifications have the potential to reduce discards without impacting their commercial catch, are almost as cheap as ordinary trammel nets, and do not need additional effort to handle. This is especially important because elasmobranch discards in SSF can be quite alarming, particularly for trammel nets, which are widely employed in the majority of coastal regions where the vulnerable juvenile stages of most elasmobranch species reside.

## Data Availability

Data used in the analyses can be accessed from the Dryad Digital Repository: https://doi.org/10.5061/dryad.z612jm6hm [45].
